# Characterization of Physicochemical Quality and Volatiles in Donkey Meat Hotpot Under Different Boiling Periods

**DOI:** 10.3390/foods14142530

**Published:** 2025-07-18

**Authors:** Lingyun Sun, Mengmeng Mi, Shujuan Sun, Lu Ding, Yan Zhao, Mingxia Zhu, Yun Wang, Muhammad Zahoor Khan, Changfa Wang, Mengmeng Li

**Affiliations:** 1 School of Agriculture and Biology, Liaocheng Research Institute of Donkey High-Efficiency Breeding and Ecological Feeding, Liaocheng University, Liaocheng 252000, China; lysun111@163.com (L.S.); dinglu0708@163.com (L.D.); 16652288780@163.com (Y.Z.); zhumingxia@lcu.edu.cn (M.Z.); wangcf1967@163.com (C.W.); 2 Liaocheng Academy of Agricultural Sciences, Liaocheng 252000, China; mi970318@163.com (M.M.); 19861210810@163.com (S.S.); lcwangyun@163.com (Y.W.)

**Keywords:** donkey meat, hotpot cooking, meat quality, gas chromatography–ion mobility spectrometry (GC-IMS), volatile compounds

## Abstract

Hotpot dishes are widely favored by consumers for their flavor profiles developed during the cooking process. This study investigated the quality characteristics and volatile compounds (VOCs) of donkey meat slices across varying boiling durations (0–42 s) using gas chromatography–ion mobility spectrometry (GC-IMS). The results demonstrated that donkey meat boiled for 12–18 s exhibited optimal characteristics in terms of meat retention, color parameters, shear force values, and pH measurements. Forty-eight distinct VOCs were identified in the samples, with aldehydes, alcohols, ketones, acids, furans, and esters representing the predominant categories. Among these compounds, 18 were identified as characteristic aroma compounds, including 3-hexanone, 2, 3-butanedione, and oct-1-en-3-ol. Samples subjected to different boiling durations were successfully differentiated through topographic plots, fingerprint mapping, and multivariate analysis. The abundance and diversity of VOCs reached peak values in samples boiled for 12–18 s. Furthermore, 28 VOCs were identified as potential markers for distinguishing between different boiling durations, including 2-butoxyethanol D, benzaldehyde D, and (E)-2-pentenal D. This study concludes that a boiling duration of 12–18 s for donkey meat during hotpot preparation yields optimal quality characteristics and volatile flavor compound profiles and provides valuable insights for standardizing cooking parameters in hotpot preparations of other meat products. It is necessary to confirm this finding with sensory evaluations in further research.

## 1. Introduction

As consumer living standards improve, heightened attention is directed towards the nutritional composition, characteristic aroma, and flavor profile of meat products. From a nutritional perspective, donkey meat demonstrates superior nutritional value compared to beef, pork, and mutton [1,2]. It contains high protein levels, polyunsaturated fatty acids (PUFAs), minerals, and vitamins [3]. Donkey meat is predominantly utilized in processed products, including donkey pancakes, spiced donkey meat, and steamed dumplings [4]. There are numerous traditional cooking methods in China, including steaming, boiling, roasting, frying, and hotpot scalding. Different cooking techniques are appropriate for various muscle parts [5]. Hotpot, such as sheep meat hotpot and beef hotpot, is a traditional Chinese fast-boiling cooking method with significant historical importance [6,7]. Nowadays, it is becoming popular to boil donkey meat slices (donkey hotpot). Thus, it is important to understand the palatability of donkey meat when using this widely popular cooking method.

Flavor constitutes a critical sensory attribute of meat, significantly influencing its palatability [8]. Volatile compounds (VOCs) contribute substantially to the characteristic aroma and flavor of cooked meats, comprising aldehydes, ketones, alcohols, acids, furans, and esters [9,10]. Previous research utilizing Orbitrap Exploris GC 240 identified numerous VOCs in mutton, including aldehydes, furans, ketones, alcohols, esters, and acids, with hexanal and 1-octen-3-ol identified as the predominant odorants in roasted mutton [11]. Aldehydes, ketones, and alcohols are identified as the main VOCs in raw donkey meat using GC-IMS [12]. The VOCs in the meat primarily originate from lipid oxidation [13], with cooking methodology and thermal exposure duration significantly affecting lipid oxidation and fatty acid compositional changes [14,15]. Hotpot cooking, a traditional Chinese cooking method, generates complex, multi-layered flavor profiles during the boiling process [16]. Hexanal and heptaldehyde have been identified as critical flavor components in instant-boil Pingling red beef [17]. Additionally, taste-related sensory characteristics, particularly “umami” and “sweet taste,” demonstrated significantly higher ratings in beef hotpot preparations compared to yakiniku (grilling) [18]. However, the VOC profile of donkey meat prepared via hotpot remains unexplored in the scientific literature.

Gas chromatography–ion mobility spectroscopy (GC–IMS) is a powerful, sensitive technique for the separation and detection of VOCs [19]. Previous investigations have employed GC-IMS to identify significant variations in VOC types and concentrations across multiple meat sources, including chicken [20], pork [21], and yak [22]. Additionally, GC-IMS combined with multivariate analysis has been utilized to identify and analyze VOCs in donkey meat from different breeds [12]. Furthermore, odor activity values (OAVs) provide a quantitative approach to assess the contributions of individual VOCs to the overall aromatic profile of meat products [23]. However, there is little research on VOCs in donkey meat hotpot. In the present study, the VOC profiles, characteristic VOCs, and differential VOCs were identified in donkey meat subjected to various boiling durations using GC–IMS, OAVs, and a multivariate analysis. This study systematically identified the physicochemical characteristics and volatile profiles of donkey meat at various time points when prepared in hotpot form, offering valuable insights for enhancing the precision and digitization of food quality control to promote the standardized production of the food industry.

## 2. Materials and Methods

### 2.1. Animals and Sample Collection

A total of 6 healthy two-year-old male Dezhou donkeys were obtained from a local farm in Liaocheng (Shandong, China). Donkeys were fed a mixed diet with 80% roughage (including corn straw and wheat straw), 15% corn, 3% soybean meal, and 2% other components. The donkeys were fed twice daily (8:30 a.m. and 4:30 p.m.) with free access to water. Donkeys were starved for 12 h, transported to a local abattoir (Shandong Dong’a Tianlong Food Co., Ltd., Liaocheng, China), and slaughtered according to international standards (CAC/RCP 41-1993 [24] and ISO/TS 34700: 2016 [25]). The donkeys were stunned using electric shocks and bled until they died. After removing the skin, the *longissimus dorsi* between the 12th and 13th ribs was immediately collected, placed on ice, and transported to the laboratory. The muscle was sliced into 1.45 ± 0.03 mm thick pieces using a slicer (CUKO, UK) and weighed. Six pieces (from six donkeys, one piece per donkey) were treated each time, and each piece was cooked separately for each time (n = 6). The meat samples were divided into six groups (T0, T6, T12, T18, T30, and T42) and boiled in a water bath at 100 °C for 0, 6, 12, 18, 30, or 42 s, respectively. After boiling, samples were reweighed for further analysis. Animal experiments were approved by the Animal Care and Use Committee of Liaocheng University (Welfare NO. 2023022706).

### 2.2. Physicochemical Properties of Meat

Referring to the operating instructions and a previous study [15], the thickness and shear force of donkey meat were determined using a Vernier caliper and a C-LM3B tenderness instrument (Northeast Agricultural University, Harbin, China), respectively. Color coordinates (lightness, L*; redness, a*; and yellowness, b*) and total color difference (ΔE) of samples were determined using a Chroma Meter CR-10Plus colorimeter (Konica Minolta Sensing Inc., Osaka, Japan) with a CIE D65 illuminant. The total color difference (ΔE) between the tested muscles was calculated using the following formula:

$$\Delta E=\sqrt{\left({\left(L*\right)}^{2}+{\left(a*\right)}^{2}+{\left(b*\right)}^{2}\right)}$$

The pH values were measured by a Mettler Toledo testo 205 pH meter (Zurich, Switzerland). The meat retention was calculated as the percentage change in the weight before and after boiling.

### 2.3. VOC Analysis

The VOCs in the donkey meat samples were analyzed based on a FlavourSpec^®^ (Gesellschaft für Analytische Sensorysteme GmbH, G.A.S., Dortmund, Germany) GC–IMS unit equipped with a capillary column (MXT-5, 15 m × 0.53 mm × 1.0 μm) and an automatic headspace sampling unit (CTC-PAL, CTC Analytics AG, Zwingen, Switzerland). A 4 g sample and 1.0 μL 2-methyl-3-heptanone (0.1 g / L) were placed into a 20 mL headspace glass bottle (Thermo, Waltham, MA, USA) and incubated at 60 °C for 15 min with spinning at 500 rpm. Subsequently, 500 μL of the headspace gas was automatically injected into the instrument. The injector temperature was set to 85 °C. The GC column temperature was 40 °C, and the carrier gas was nitrogen with purity ≥ 99.999%. Carrier gas flow rate from 0 to 2 min was 2 mL/min; the rate from 2 to 10 min was 2–20 mL/min; and the rate from 10 to 20 min was 20–100 mL/min. The drift tube of the IMS instrument was 9.8 cm long, and the drift temperatures were 60 °C and 45 °C, respectively. The drift tube voltage was set to 5 kV. The drift gas was nitrogen with purity ≥ 99.999%, and the flow rate was 150 mL/min. 3H ionization was performed in positive ion mode.

### 2.4. VOC Identification

The VOCs’ retention indices (RIs) were compared with those of C4–C9 n-ketones (Sinopharm Chemical Reagent Beijing Co., Ltd., Beijing, China) obtained under the same analytical conditions. The VOCs were identified using the RIs and drift times (DTs) of the standards in the NIST (National Institute of Standards and Technology, Gaithersburg, MD, USA) 2014 library and GC–IMS database (G.A.S., Dortmund, Germany). The ratio of concentration to threshold was used to determine the OAV. The key characteristic VOCs were defined as those with OAV ≥ 1, with higher values indicating greater contributions to the overall flavor. The formula for OAV is as follows:

OAVi = Ci/Ti × 100, where Ci and Ti are represented as the absolute content (μg/kg) and threshold (μg/kg) for each VOC, respectively.

### 2.5. Statistical Analysis

Data were analyzed by one-way ANOVA and Duncan’s test using SPSS 24.0 (SPSS Inc., Chicago, IL, USA), and the mean ± standard error of the mean (SEM) was expressed. Differences among the groups were considered significant at *p* < 0.05. The spectra and fingerprints were processed using the Reporter plug-in and Gallery Plot plug-in, respectively. Principal component analysis (PCA), partial least squares discriminant analysis (PLS-DA), orthogonal PLS-DA (OPLS-DA), and heatmap analysis were performed using MetaboAnalyst 5.0 online software. Differential VOCs were determined according to a variable importance in projection (VIP) score of >1 and *p* < 0.05. GraphPad Prism 10.0 (GraphPad Software, Inc, San Diego, CA, USA.) was used to create pie charts and histograms.

## 3. Results

### 3.1. Physicochemical Characteristics of Donkey Meat Boiled for Different Lengths of Time

As shown in Table 1, the meat retention rate decreased significantly with the boiling time from T0 to T30 (*p* < 0.05), and the meat retention rates were similar in the T30 and T42 groups (*p* > 0.05). The total color difference (ΔE) and L* value were lower in the T6, T12, T18, and T30 groups than in the T0 group, and they were the lowest in the T42 group (*p* < 0.05). The a* value decreased significantly with the boiling time from the T0 to T30 groups, except for the T18 group (*p* < 0.05). The b* values were lower in the T12 and T18 groups than in the T6 group, but higher than those of the T0, T30, and T42 groups (*p* < 0.05). The shear force values of meat in the T12 and T18 groups were lower than those in the T30 and T42 groups, whereas they were higher than those of the T0 group (*p* < 0.05). The pH value was lower in the T12, T18, and T30 groups than in the T42 group, whereas it was higher than those of the T0 and T6 groups (*p* < 0.05).

### 3.2. VOC Profiles of Donkey Meat

**Figure 1:**
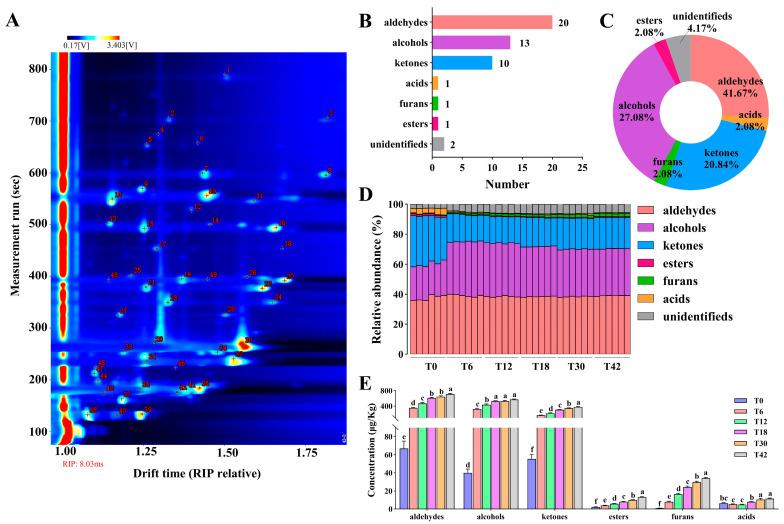
VOC profiles of donkey meat boiled for 0–42s. Signal of VOCs (**A**). Number (**B**) and percentage (**C**) of VOC classes. Abundance (**D**) and concentrations (**E**) of VOC classes. Data presented as mean ± SEM (n = 6). Different small letters indicate significant differences among groups (*p* < 0.05). VOC, volatile compound. T0, T6, T12, T18, T30, and T42 indicate 0, 6, 12, 18, 30, and 42 s.

A total of 48 VOCs were detected in the donkey meat (Figure 1A and Table 2), including 20 aldehydes, 13 alcohols, 10 ketones, one acid, one furan, one ester, and two unidentified components (Figure 1B), i.e., 41.67% aldehyde, 27.08% alcohol, 20.83% ketone, 2.08% acid, 2.08% furan, 2.08% ester, and 4.17% unidentified (Figure 1C). Aldehydes are the most abundant VOCs, followed by alcohols and ketones, in the donkey meat (Figure 1D). The concentrations of ketones, esters, and furans increased significantly with the boiling time from the T0 to T42 groups (*p* < 0.05; Figure 1E). The concentrations of aldehydes and alcohols increased significantly with the boiling time from the T0 to T18 groups (*p* < 0.05), and there was no significant change except for the aldehydes in the other groups (*p* > 0.05; Figure 1E). The acid concentration of the meat in the T18 group was higher than in the T6 and T12 groups (*p* < 0.05), whereas it was lower than that in the T30 and T42 groups (*p* < 0.05; Figure 1E).

### 3.3. Difference in VOCs for the Different Boiling Times

**Figure 2:**
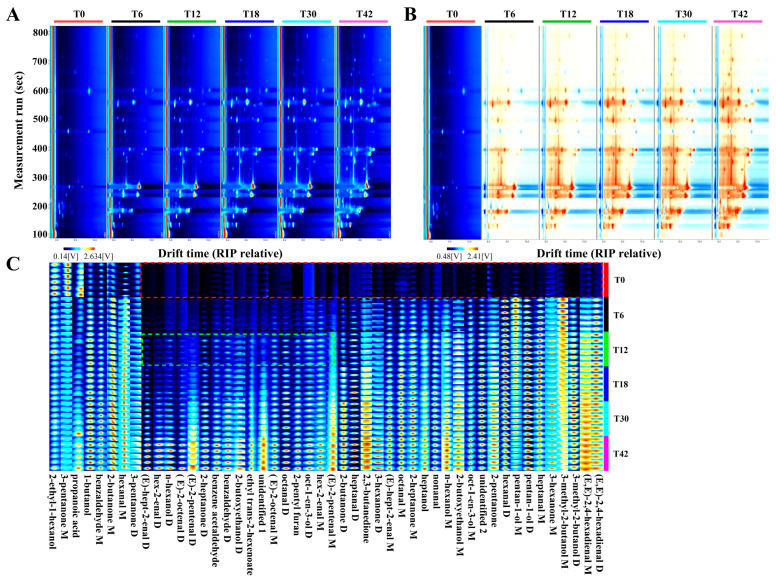
Comparison of the VOCs in donkey meat boiled for 0–42 s. Topographic representations of the spectra (**A**), difference spectra (**B**), and fingerprints of gallery plots (**C**) for VOCs. The brighter the color of the signal peak, the higher the concentration of the component. VOC, volatile compound. T0, T6, T12, T18, T30, and T42 indicate 0, 6, 12, 18, 30, and 42 s.

**Figure 3:**
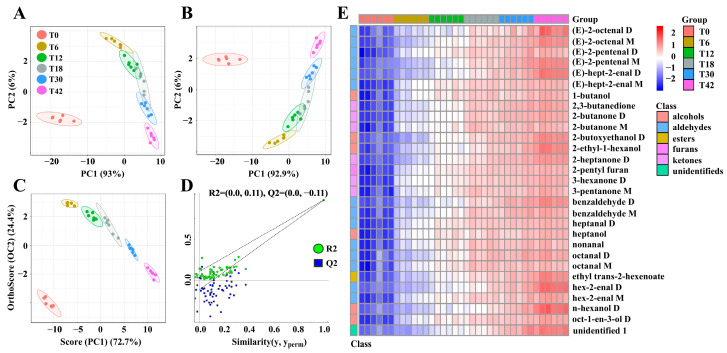
Differential VOCs in donkey meat boiled for 0–42 s. Principal component analysis (PCA) (**A**), partial least squares discriminant analysis (PLS-DA) (**B**), and OPLS-DA (**C**) score plots based on VOC data (R2X = 0.992, R2Y = 0.991, Q2 = 0.988). Corresponding OPLS-DA validation plots (**D**). Heatmap of VOCs in donkey meats identified using VIP score > 1 and *p* < 0.05 (**E**). VOC, volatile compound. T0, T6, T12, T18, T30, and T42 indicate 0, 6, 12, 18, 30, and 42 s.

According to the topographic plots, good repeatability was shown, and a significant difference was observed between the fingerprints at different times (Figure 2A,B). In addition, the contents of 2-butoxyethanol D, benzaldehyde D, benzene acetaldehyde, 2-heptanone D, (E)-2-pentenal D, (E)-2-octenal D, n-hexanol D, hex-2-enal D, and (E)-hept-2-enal D were increased with the extension of the boiling time (Figure 2C). As shown in Figure 3A, B, the VOCs in the meat boiling from the T0 to T42 groups could be discriminated from each other by using a PCA and PLS-DA (except for the T18 group). However, the VOCs in the T18 group were well differentiated from those in the T0, T6, and T42 groups. The OPLS-DA analysis could discriminate differential VOCs from the T0 to T42 groups (Figure 3C), and the OPLS-DA validation plots showed that it was robust and that overfitting did not occur (Figure 3D). A total of 28 differential VOCs belonging to six classes in the meat were identified, setting the VIP score > 1 and *p* < 0.05 (Figure 3E and Table 3), including 14 aldehydes, six alcohols, six ketones, one furan, and one ester. The concentrations of benzaldehyde D, (E)-2-octenal M, heptanal D, (E)-hept-2-enal M, 2-butoxyethanol D, 2-ethyl-1-hexanol, oct-1-en-3-ol D, 2,3-butanedione, 2-butanone D, 3-hexanone D, 2-pentyl furan, and ethyl trans-2-hexenoate increased significantly with the boiling time from the T0 to T42 groups (*p* < 0.05). In addition, from the T0 to T18 groups, the concentration of nonanal, octanal M, and 2-butanone M increased significantly (*p* < 0.05), and the concentration of octanal D, benzaldehyde M, and (E)-2-pentenal D increased significantly with the boiling time from the T0 to T30 groups (*p* < 0.05, Figure 3E and Table 3).

### 3.4. The Characteristic Aroma Compounds in Boiled Donkey Meat

As shown in Table 4, a total of 18 characteristic aroma VOCs with OAV > 1 were identified in the donkey meat. The OAV of 3-hexanone is the largest (OAV = 89.09–540.24), followed by 2,3-butanedione (OAV = 20.65–210.70) and oct-1-en-3-ol (OAV = 9.28–129.82). The characteristic aroma compounds were further divided into four classes, including nine aldehydes, four alcohols, four ketones, and one furan. The OAVs of (E)-2-octenal, (E)-hept-2-enal, 3-hexanone, 2,3-butanedione, and 2-pentylfuran increased significantly with the boiling time from the T0 to T42 groups (*p* < 0.05). The OAVs of nonanal, octanal, hexanal, (E, E)-2,4-hexadienal, and pentan-1-ol increased significantly with the boiling time from the T0 to T18 groups, and the highest OAV was in the T18 group (*p* < 0.05; Table 4).

## 4. Discussion

The sensory characteristics of meat, particularly color, juiciness, and tenderness, play crucial roles in determining meat quality [26]. A significant factor affecting these properties is cooking loss, which encompasses the release of liquid and soluble matter during the cooking process, ultimately influencing meat juiciness [27]. In the present study, the donkey meat retention rate exhibited an inverse relationship with the boiling duration, aligning with previous findings of duck meat studies [28]. Furthermore, these results demonstrated that all color parameters were significantly influenced by the boiling duration. Specifically, the L* values of donkey meat showed a decreasing trend with extended boiling times, in parallel with observations in braised squab skin [29]. This phenomenon can be attributed to two primary mechanisms: firstly, the enhanced cooking loss facilitates muscle fiber exposure and subsequent light scattering [30]; and secondly, the increased denaturation and aggregation of sarcoplasmic and myofibrillar proteins contribute to elevated light scattering [31]. Myoglobin (Mb), a heme-containing globular protein, regulates meat coloration through the modulation of the iron center’s redox state and the specific ligands bound to its coordination site [32]. And the variations in a* and b* values were predominantly associated with the oxidative conversion of ferrous Mb to ferric myoglobin during the boiling process [33]. Regarding texture properties, the shear force serves as a direct indicator of a meat product’s mouthfeel, with lower values corresponding to superior chewing quality and meat quality [34]. These findings revealed an upward trend in shear force values, with the optimal tenderness observed between 12 s and 18 s of boiling, consistent with previous studies on boiled rabbit meat, which indicated that the oxidation, denaturation, aggregation, and cross-linking of proteins led to an increase in shear force [35,36]. Moreover, pH, a critical indicator of meat quality that significantly influences tenderness [37], exhibited higher values compared to raw meat and demonstrated an increasing trend with extended boiling times. This observation aligns with findings from female carabeef meat studies [38] and can be attributed to the simultaneous loss of lactic acid and water during cooking, as well as the disruption of the protein structure’s chemical bonds, which results in the burial of muscle protein acid groups [39].

In terms of VOCs, which serve as essential biomarkers providing valuable information about meat quality [40], the study identified 48 distinct VOCs in donkey meat, correlating with previous research that detected 40 VOCs using GC-IMS analysis [41]. These compounds were predominantly categorized into aldehydes, alcohols, ketones, acids, furans, and esters, with aldehydes representing the most abundant class, followed by alcohols and ketones. This distribution pattern is consistent with previous findings in boiled donkey meat [10], with concentrations showing a positive correlation with the boiling duration, likely due to enhanced lipid oxidative degradation.

The GC-IMS spectra and fingerprint analysis proved to be effective tools for visual discrimination among samples [19]. The investigation revealed significant spectral variations among donkey meat samples subjected to different boiling durations, which were further validated through their VOC fingerprints. The donkey meat slices of the T0 group were the same as the raw donkey meat. Notable differences were observed in compounds such as (E)-2-pentenal D, (E)-2-octenal D, and n-hexanol D, consistent with previous findings in studies examining different donkey meat cuts [41]. To further discriminate the VOCs of donkey meat with different boiling times, a multivariate analysis and heatmap visualization were applied to analyze the GC-IMS data. PCA is a multivariate statistical analysis technique that aids in the visualization of similarities and differences. The cumulative contribution rate represents the predominant flavor characteristics of the sample, and the closer the distance between the samples, the higher the similarity between their aroma components and relative content [21]. Furthermore, the PLS-DA and OPLS-DA analyses, serving as supervised discriminant analysis methods, effectively distinguished observations between groups [20]. In this study, these multivariate analysis methods identified differences in the VOCs between the T0, T6, T12, T18, T30, and T42 groups. Moreover, the OPLS-DA validation plots showed that it was robust and that overfitting did not occur. VIP values can be used to reflect the contribution of VOCs to the overall flavor during boiling, with higher VIP values of VOCs indicating greater contributions to meat flavor [42]. The application of these multivariate analysis methods (PCA, PLS-DA, OPLS-DA, and heatmap analysis) confirmed the reliability of the data and fingerprints obtained through GC-IMS analysis, aligning with findings from studies on donkey meat of different breeds [12].

Previous research has established that VOC profiles in meat are significantly influenced by both cooking methods and duration [17], a finding documented by our observation of boiling time-dependent VOC variations in donkey meat. Comparative studies have identified key VOCs in various meat products under different cooking conditions. For instance, benzaldehyde, hexanol, 1-heptanol, 1-octanol, (E)-2-octenal, and 2-pentyl furan were identified as key VOCs in pork roasted for different lengths of time [43], while 1-octen-3-ol, decanal, nonanal, and octanal were found to differ according to boiling times in crayfish meat [44]. Heptanal, benzaldehyde, 1-heptanol, 2, 3-butanedione, 2-butanone-M, 2-heptanone, and ethyl acetate-D were correlated with steam times in pork belly [21]. This study identified several characteristic VOCs associated with different boiling durations, including benzaldehyde D, (E)-2-octenal M, heptanal D, (E)-hept-2-enal M, 2-butoxyethanol M, 2-ethyl-1-hexanol, 2,3-butanedione, 2-butanone D, 3-hexanone D, 2-pentyl furan, and ethyl trans-2-hexenoate. The formation of these compounds can be attributed to lipid degradation processes, which generate aldehydes, alcohols, hydrocarbons, and ketones that contribute significantly to aroma development [45]. An extended cooking duration has been shown to enhance lipid oxidation [15], with studies demonstrating positive correlations between specific VOC concentrations (such as hexanal, nonanal, and 1-octen-3-ol) and lipid concentrations in mutton subjected to different roasting times [11]. Recent investigations have confirmed that the lipid content in donkey meat is significantly affected by the boiling duration [10], suggesting that VOC content undergoes dynamic changes throughout the boiling process.

The contribution of individual VOCs to overall flavor is determined by their concentration and odor threshold [46], with OAVs showing a positive correlation to the contribution of VOCs to the flavor profile [42]. Among the 18 aroma compounds identified in this study, aldehydes emerged as the predominant contributors to boiled donkey meat aroma, followed by alcohols, ketones, and furans. This finding adds to the growing body of research on characteristic meat flavors, which has identified various key odorants across different meat products and cooking methods [23]. For instance, nonanal, octanal, heptanal, hexanal, and oct-1-en-3-ol were reported as key odorants in Beijing roasted ducks [47]. Pentanal, hexanal, octanal, nonanal, and (E)-2-octenal were shown to have a significant effect on flavor in boiled beef because of their grassy, citrusy, and fatty odor [48]. (E, E)-2, 4-hexadienal, 1-hexanol, and 2-pentyl-furan were reported to be very important for rabbit meat cooked using different methods [49]. 3-methylbutanal, 2, 3-butanedione, 1-octen-3-one, and (E, Z)-2, 6-nonadienal were identified as predominant odorants in the steam-cooked tail meat of American lobster [50]. Recent studies have shown that hexanal, heptanal, octanal, and 1-octene-3-ol are the main VOCs in boiled donkey meat using an electronic nose analysis [51]. Previous studies have indicated that most C6-C10 aliphatic aldehydes produce green and fatty odor notes [23]. Thus, these characteristic aroma compounds are the main contributors to the aroma of donkey meat hotpots. However, this study still has certain limitations. For example, this study only measured the physicochemical indicators such as L*, a*, and b*. There was a lack of precise sensory tests. Even though the tasting was conducted after the boiling of donkey meat, the tasting results were consistent with the test results. However, the data were not precise enough, and the conclusion was not objective enough. Future studies should conduct sensory evaluations to relate our results to consumers’ experiences for added reference value.

## 5. Conclusions

This study comprehensively analyzed and compared the quality and VOCs of donkey meat subjected to different boiling times. The optimal boiling time was identified as 12–18 s, which yielded superior results in terms of meat retention, color parameters, shear force, and pH levels. The analysis identified a total of 48 VOCs across the donkey meat samples. A total of 28 differential VOCs were identified among the donkey meat samples from the different boiling treatments, including 2-butoxyethanol, benzaldehyde, and (E)-2-pentenal. The samples boiled for 12–18 s demonstrated significantly greater numbers and concentrations of VOCs. Among these, 18 key aroma-contributing VOCs were consistently detected, including 3-hexanone, 2,3-butanedione, and oct-1-en-3-ol. In conclusion, boiling the donkey meat for 12–18 s to prepare the hotpot optimized both its quality parameters and volatile flavor compounds. This result could be used to enhance the precision and digitization of food quality control and promote the standardized production of the food industry. It is necessary to confirm this finding with sensory evaluations in further research.

## Figures and Tables

**Table 1:** Physicochemical characteristics of donkey meat boiled for 0–42 s.

Item.	T0	T6	T12	T18	T30	T42	*p* Value
Meat retention rate (%)	100.00 ± 0.00 ^a^	80.24 ± 1.37 ^b^	75.88 ± 1.27 ^c^	72.38 ± 1.19 ^d^	66.08 ± 0.52 ^e^	65.33 ± 0.39 ^e^	0.0001
ΔE	54.92 ± 0.32 ^a^	39.65 ± 0.43 ^b^	38.80 ± 0.85 ^b^	38.72 ± 0.41 ^b^	39.39 ± 0.32 ^b^	35.85 ± 0.63 ^c^	0.0001
Luminosity (L*)	51.14 ± 0.37 ^a^	36.28 ± 0.52 ^b^	36.26 ± 0.96 ^b^	36.34 ± 0.55 ^b^	37.52 ± 0.38 ^b^	33.83 ± 0.71 ^c^	0.0001
Redness (a*)	18.34 ± 0.60 ^a^	9.48 ± 0.33 ^b^	7.23 ± 0.35 ^c^	6.43 ± 0.35 ^cd^	5.40 ± 0.12 ^d^	5.58 ± 0.12 ^d^	0.0001
Yellowness (b*)	7.65 ± 0.38 ^d^	12.74 ± 0.25 ^a^	11.56 ± 0.23 ^b^	11.57 ± 0.27 ^b^	10.66 ± 0.18 ^c^	10.39 ± 0.26 ^c^	0.0001
Shear force (N)	6.72 ± 0.71 ^c^	24.11 ± 1.22 ^ab^	21.40 ± 1.03 ^b^	21.02 ± 1.25 ^b^	26.37 ± 1.42 ^a^	27.79 ± 2.33 ^a^	0.0001
pH	5.78 ± 0.03 ^d^	6.06 ± 0.06 ^c^	6.35 ± 0.04 ^b^	6.27 ± 0.01 ^b^	6.32 ± 0.03 ^b^	6.49 ± 0.05 ^a^	0.0001

ΔE, total color difference. Data presented as mean ± SEM (n = 6); different letters in each parameter indicate significant differences at *p* < 0.05.

**Table 2:** Information on VOCs in donkey meat.

No.	Compound	CAS #	Formula	MW	RI	Rt (s)	Dt (a.u.)	Comment
1	nonanal	C124196	C_9_H_18_O	142.2	1107.2	789.931	1.50274	
2	(E)-2-octenal M	C2548870	C_8_H_14_O	126.2	1068.9	706.209	1.32849	Monomer
3	(E)-2-octenal D	C2548870	C_8_H_14_O	126.2	1068.9	706.209	1.8197	Dimer
4	ethyl trans-2-hexenoate	C27829727	C_8_H_14_O_2_	142.2	1053	674.184	1.29699	
5	benzene acetaldehyde	C122781	C_8_H_8_O	120.2	1043.2	655.208	1.259	
6	2-ethyl-1-hexanol	C104767	C_8_H_18_O	130.2	1044.3	657.241	1.41826	
7	octanal M	C124130	C_8_H_16_O	128.2	1012.5	598.956	1.43287	Monomer
8	octanal D	C124130	C_8_H_16_O	128.2	1010.2	594.89	1.81568	Dimer
9	2-pentyl furan	C3777693	C_9_H_14_O	138.2	995.4	569.136	1.24731	
10	oct-1-en-3-ol M	C3391864	C_8_H_16_O	128.2	986.3	548.484	1.1542	Monomer
11	oct-1-en-3-ol D	C3391864	C_8_H_16_O	128.2	985.6	547.053	1.59344	Dimer
12	heptanol	C53535334	C_7_H_16_O	116.2	976	526.306	1.39994	
13	benzaldehyde M	C100527	C_7_H_6_O	106.1	964.3	501.981	1.13872	Monomer
14	benzaldehyde D	C100527	C_7_H_6_O	106.1	963.2	499.835	1.45605	Dimer
15	(E)-hept-2-enal M	C18829555	C_7_H_12_O	112.2	959.6	492.497	1.25348	Monomer
16	(E)-hept-2-enal D	C18829555	C_7_H_12_O	112.2	960.4	494.221	1.66176	Dimer
17	heptanal M	C111717	C_7_H_14_O	114.2	904.8	394.863	1.36941	Monomer
18	heptanal D	C111717	C_7_H_14_O	114.2	902.3	390.924	1.68703	Dimer
19	2-heptanone M	C110430	C_7_H_14_O	114.2	894.7	379.104	1.25789	Monomer
20	2-heptanone D	C110430	C_7_H_14_O	114.2	893.1	376.74	1.61786	Dimer
21	n-hexanol M	C111273	C_6_H_14_O	102.2	872.1	348.939	1.32652	Monomer
22	n-hexanol D	C111273	C_6_H_14_O	102.2	873.8	351.173	1.6497	Dimer
23	2-butoxyethanol M	C111762	C_6_H_14_O_2_	118.2	909.9	403.128	1.21417	Monomer
24	2-butoxyethanol D	C111762	C_6_H_14_O_2_	118.2	907.1	398.659	1.57121	Dimer
25	hex-2-enal M	C505577	C_6_H_10_O	98.1	852	324.457	1.1715	Monomer
26	hex-2-enal D	C505577	C_6_H_10_O	98.1	855	327.915	1.50489	Dimer
27	hexanal M	C66251	C_6_H_12_O	100.2	801.3	269.887	1.28113	Monomer
28	hexanal D	C66251	C_6_H_12_O	100.2	798.9	267.572	1.55918	Dimer
29	pentan-1-ol M	C71410	C_5_H_12_O	88.1	765.3	235.94	1.25998	Monomer
30	pentan-1-ol D	C71410	C_5_H_12_O	88.1	770.3	240.57	1.52958	Dimer
31	3-hexanone M	C589388	C_6_H_12_O	100.2	785.6	254.988	1.18776	Monomer
32	3-hexanone D	C589388	C_6_H_12_O	100.2	784	253.485	1.47976	Dimer
33	3-methyl-2-butanol M	C598754	C_5_H_12_O	88.1	698	182.442	1.24044	Monomer
34	3-methyl-2-butanol D	C598754	C_5_H_12_O	88.1	700.4	184.115	1.42477	Dimer
35	1-butanol	C71363	C_4_H_10_O	74.1	664.5	163.155	1.18342	
36	2-butanone M	C78933	C_4_H_8_O	72.1	599.1	132.777	1.0779	Monomer
37	2-butanone D	C78933	C_4_H_8_O	72.1	602.3	134.117	1.24701	Dimer
38	2,3-butanedione	C431038	C_4_H_6_O_2_	86.1	595.1	131.138	1.16882	
39	2-pentanone	C107879	C_5_H_10_O	86.1	696.8	181.634	1.38354	
40	3-pentanone M	C96220	C_5_H_10_O	86.1	687.1	175.18	1.12887	Monomer
41	3-pentanone D	C96220	C_5_H_10_O	86.1	689.6	176.699	1.35483	Dimer
42	propanoic acid	C79094	C_3_H_6_O_2_	74.1	720.4	198.75	1.10936	
43	(E)-2-pentenal M	C1576870	C_5_H_8_O	84.1	751.4	223.787	1.10304	Monomer
44	(E)-2-pentenal D	C1576870	C_5_H_8_O	84.1	750.8	223.223	1.35021	Dimer
45	unidentified 1	-	-	-	737.6	212.232	1.09812	
46	(E, E)-2,4-hexadienal M	C142836	C_6_H_8_O	96.1	904	393.653	1.14235	Monomer
47	(E, E)-2,4-hexadienal D	C142836	C_6_H_8_O	96.1	904.3	394.192	1.4496	Dimer
48	unidentified 2	-	-	-	989.2	554.965	1.44706	

CAS#: CAS Registry Number. RI, Retention index. Dt, Drift time. MW, Molecular weight. Rt, Retention time.

**Table 3:** VOC content of donkey meat boiled for 0–42 s (μg/kg).

No.	Compound	Class	T0	T6	T12	T18	T30	T42	*p* Value	VIP
1	hex-2-enal D	aldehydes	0.56 ± 0.04 ^e^	1.11 ± 0.06 ^de^	1.98 ± 0.14 ^d^	3.19 ± 0.34 ^c^	5.24 ± 0.37 ^b^	10.12 ± 0.53 ^a^	0.0000	1.177
2	(E)-hept-2-enal D	aldehydes	2.69 ± 0.29 ^e^	5.94 ± 0.47 ^e^	10.60 ± 0.78 ^d^	18.75 ± 1.84 ^c^	28.24 ± 2.04 ^b^	51.87 ± 2.53 ^a^	0.0000	1.174
3	(E)-2-pentenal M	aldehydes	1.13 ± 0.13 ^e^	2.21 ± 0.17 ^e^	3.72 ± 0.31 ^d^	6.75 ± 0.42 ^c^	10.07 ± 0.65 ^b^	13.90 ± 0.45 ^a^	0.0000	1.165
4	benzaldehyde D	aldehydes	1.45 ± 0.12 ^f^	3.00 ± 0.18 ^e^	4.12 ± 0.24 ^d^	6.39 ± 0.28 ^c^	8.71 ± 0.35 ^b^	10.69 ± 0.37 ^a^	0.0000	1.150
5	(E)-2-octenal M	aldehydes	1.31 ± 0.15 ^f^	3.11 ± 0.26 ^e^	5.83 ± 0.36 ^d^	8.44 ± 0.61 ^c^	10.89 ± 0.62 ^b^	15.85 ± 0.66 ^a^	0.0000	1.150
6	(E)-2-octenal D	aldehydes	1.57 ± 0.12 ^d^	2.65 ± 0.09 ^c^	2.98 ± 0.16 ^c^	3.98 ± 0.17 ^b^	4.62 ± 0.30 ^b^	6.32 ± 0.37 ^a^	0.0000	1.130
7	octanal D	aldehydes	2.03 ± 0.50 ^e^	4.72 ± 0.31 ^d^	9.07 ± 0.76 ^c^	15.10 ± 1.28 ^b^	17.85 ± 0.88 ^a^	19.69 ± 0.83 ^a^	0.0000	1.099
8	hex-2-enal M	aldehydes	2.34 ± 0.19 ^e^	6.77 ± 0.49 ^d^	9.66 ± 0.57 ^c^	12.67 ± 0.47 ^b^	14.06 ± 0.91 ^b^	16.88 ± 0.64 ^a^	0.0000	1.076
9	benzaldehyde M	aldehydes	5.72 ± 0.52 ^e^	12.52 ± 0.53 ^d^	15.80 ± 0.76 ^c^	21.60 ± 0.51 ^b^	24.35 ± 0.88 ^a^	24.73 ± 0.59 ^a^	0.0000	1.071
10	heptanal D	aldehydes	2.15 ± 0.18 ^f^	15.24 ± 1.34 ^e^	29.18 ± 1.69 ^d^	41.19 ± 1.64 ^c^	47.03 ± 1.48 ^b^	52.49 ± 1.37 ^a^	0.0000	1.033
11	nonanal	aldehydes	4.06 ± 0.37 ^d^	10.60 ± 0.62 ^c^	15.46 ± 0.88 ^b^	19.53 ± 0.90 ^a^	20.38 ± 0.59 ^a^	20.71 ± 0.68 ^a^	0.0000	1.030
12	(E)-2-pentenal D	aldehydes	0.39 ± 0.05 ^e^	1.58 ± 0.14 ^d^	2.40 ± 0.13 ^c^	3.10 ± 0.09 ^b^	3.77 ± 0.26 ^a^	3.83 ± 0.15 ^a^	0.0000	1.029
13	(E)-hept-2-enal M	aldehydes	2.26 ± 0.22 ^f^	17.98 ± 1.89 ^e^	30.30 ± 1.99 ^c^	41.89 ± 1.95 ^b^	46.66 ± 2.54 ^b^	55.36 ± 1.81 ^a^	0.0000	1.020
14	octanal M	aldehydes	5.97 ± 1.23 ^d^	17.89 ± 1.20 ^c^	28.48 ± 1.54 ^b^	39.68 ± 1.49 ^a^	40.41 ± 1.53 ^a^	41.17 ± 1.07 ^a^	0.0000	1.016
15	n-hexanol D	alcohols	1.98 ± 0.18 ^d^	3.48 ± 0.21 ^d^	5.81 ± 0.49 ^c^	7.32 ± 0.59 ^c^	12.5 ± 0.89 ^b^	23.40 ± 1.31 ^a^	0.0000	1.172
16	2-butoxyethanol D	alcohols	0.99 ± 0.09 ^f^	1.75 ± 0.17 ^e^	3.64 ± 0.15 ^d^	5.34 ± 0.34 ^c^	6.84 ± 0.18 ^b^	9.59 ± 0.25 ^a^	0.0000	1.160
17	2-ethyl-1-hexanol	alcohols	0.98 ± 0.10 ^f^	2.42 ± 0.21 ^e^	3.68 ± 0.25 ^d^	5.22 ± 0.38 ^c^	7.10 ± 0.46 ^b^	10.85 ± 0.51 ^a^	0.0000	1.151
18	oct-1-en-3-ol D	alcohols	2.92 ± 0.25 ^f^	10.77 ± 0.97 ^e^	18.70 ± 1.33 ^d^	23.85 ± 1.31 ^c^	27.45 ± 0.97 ^b^	33.61 ± 1.03 ^a^	0.0000	1.077
19	1-butanol	alcohols	9.91 ± 1.02 ^e^	25.88 ± 1.93 ^d^	37.39 ± 2.05 ^c^	44.56 ± 1.40 ^b^	43.86 ± 1.68 ^b^	49.98 ± 1.26 ^a^	0.0000	1.022
20	heptanol	alcohols	0.92 ± 0.09 ^e^	4.25 ± 0.39 ^d^	6.22 ± 0.42 ^c^	7.00 ± 0.50 ^bc^	7.51 ± 0.36 ^b^	9.11 ± 0.25 ^a^	0.0000	1.004
21	2-heptanone D	ketones	1.42 ± 0.14 ^e^	5.49 ± 0.73 ^e^	14.70 ± 1.35 ^d^	24.02 ± 2.39 ^c^	37.19 ± 1.56 ^b^	53.94 ± 1.59 ^a^	0.0000	1.146
22	2,3-butanedione	ketones	3.72 ± 0.35 ^f^	12.18 ± 0.40 ^e^	17.90 ± 0.83 ^d^	28.49 ± 0.60 ^c^	34.55 ± 1.10 ^b^	37.93 ± 1.07 ^a^	0.0000	1.089
23	2-butanone D	ketones	2.37 ± 0.19 ^f^	15.81 ± 0.55 ^e^	25.27 ± 0.96 ^d^	50.83 ± 1.50 ^c^	68.79 ± 1.46 ^b^	76.73 ± 2.97 ^a^	0.0000	1.079
24	3-pentanone M	ketones	5.31 ± 0.43 ^e^	11.86 ± 0.48 ^d^	15.21 ± 0.70 ^c^	18.57 ± 0.33 ^b^	19.19 ± 0.48 ^b^	20.75 ± 0.55 ^a^	0.0000	1.034
25	3-hexanone D	ketones	1.89 ± 0.15 ^f^	10.07 ± 0.51 ^e^	13.83 ± 0.60 ^d^	18.12 ± 0.56 ^c^	21.29 ± 0.59 ^b^	25.31 ± 0.59 ^a^	0.0000	1.031
26	2-butanone M	ketones	17.40 ± 1.58 ^d^	46.08 ± 1.70 ^c^	56.36 ± 2.58 ^b^	76.05 ± 1.79 ^a^	79.22 ± 2.75 ^a^	81.20 ± 2.12 ^a^	0.0000	1.017
27	2-pentyl furan	furan	0.88 ± 0.12 ^f^	8.04 ± 0.83 ^e^	16.40 ± 1.10 ^d^	24.29 ± 1.18 ^c^	29.57 ± 1.20 ^b^	34.02 ± 1.00 ^a^	0.0000	1.040
28	ethyl trans-2-hexenoate	ester	2.14 ± 0.24 ^f^	4.08 ± 0.22 ^e^	5.99 ± 0.36 ^d^	8.24 ± 0.40 ^c^	9.97 ± 0.45 ^b^	13.32 ± 0.47 ^a^	0.0000	1.147

VOC, volatile compound. VIP, variable importance in projection score. Data are presented as mean ± SEM (n = 6); different small letters indicate significant differences among groups (*p* < 0.05).

**Table 4:** The OAVs of donkey meat boiled for 0–42s.

No.	Compound	Class	Thresholds (μg/kg)	Odor	OAVs					
					T0	T6	T12	T18	T30	T42
1	nonanal	aldehydes	1.10	green, citrusy, waxy, sweet	3.69 ± 0.33 ^d^	9.64 ± 0.56 ^c^	14.05 ± 0.80 ^b^	17.75 ± 0.82 ^a^	18.52 ± 0.54 ^a^	18.82 ± 0.62 ^a^
2	(E)-2-octenal	aldehydes	3.00	green, jasmine, mint, bitter,	0.96 ± 0.09 ^f^	1.92 ± 0.11 ^e^	2.94 ± 0.16 ^d^	4.14 ± 0.24 ^c^	5.17 ± 0.30 ^b^	7.39 ± 0.34 ^a^
3	benzene acetaldehyde	aldehydes	4.00	sweet, honey-flavored	1.29 ± 0.14 ^b^	1.51 ± 0.08 ^ab^	1.6 ± 0.09 ^a^	1.69 ± 0.04 ^a^	1.61 ± 0.09 ^a^	1.43 ± 0.04 ^ab^
4	octanal	aldehydes	0.59	green, citrusy, lemony, fatty,	13.56 ± 2.92 ^d^	38.34 ± 2.54 ^c^	63.65 ± 3.82 ^b^	92.85 ± 4.62 ^a^	98.74 ± 3.76 ^a^	103.17 ± 3.08 ^a^
5	benzaldehyde	aldehydes	6.40	nutty, almond-like, like burnt sugar	1.12 ± 0.1 ^e^	2.42 ± 0.11 ^d^	3.11 ± 0.15 ^c^	4.37 ± 0.11 ^b^	5.17 ± 0.19 ^a^	5.53 ± 0.15 ^a^
6	(E)-hept-2-enal	aldehydes	3.00	green, pungent, fatty	1.65 ± 0.17 ^f^	7.97 ± 0.78 ^e^	13.63 ± 0.92 ^d^	20.21 ± 1.24 ^c^	24.97 ± 1.52 ^b^	35.74 ± 1.40 ^a^
7	heptanal	aldehydes	2.80	green, jasmine, mint, oily	1.84 ± 0.19 ^e^	15.26 ± 1.06 ^d^	23.75 ± 1.16 ^c^	31.23 ± 0.88 ^b^	32.97 ± 1.05 ^ab^	34.72 ± 0.82 ^a^
8	hexanal	aldehydes	4.50	green, oily	4.92 ± 0.72 ^d^	43.92 ± 1.76 ^c^	52.17 ± 2.45 ^b^	64.35 ± 1.19 ^a^	63.78 ± 2.02 ^a^	65.13 ± 1.69 ^a^
9	(E, E)-2,4-hexadienal	aldehydes	1.80	green, floral, sweet, citrusy, spicy	1.66 ± 0.15 ^d^	8.28 ± 0.43 ^c^	10.97 ± 0.58 ^b^	12.91 ± 0.34 ^a^	12.12 ± 0.52 ^ab^	11.89 ± 0.32 ^ab^
10	oct-1-en-3-ol	alcohols	1.00	mushroom-like, potato-like, smoky	9.28 ± 0.95 ^e^	58.88 ± 5.06 ^d^	92.00 ± 5.54 ^c^	110.68 ± 4.33 ^b^	118.35 ± 4.25 ^ab^	129.82 ± 3.24 ^a^
11	heptanol	alcohols	5.40	green, floral, woody, oily	0.17 ± 0.02 ^e^	0.79 ± 0.07 ^d^	1.15 ± 0.08 ^c^	1.30 ± 0.09 ^bc^	1.39 ± 0.07 ^b^	1.69 ± 0.05 ^a^
12	n-hexanol	alcohols	4.95	green, woody, fatty, fruity	0.66 ± 0.06 ^e^	3.19 ± 0.28 ^d^	5.07 ± 0.36 ^c^	5.93 ± 0.26 ^c^	7.88 ± 0.49 ^b^	11.10 ± 0.46 ^a^
13	pentan-1-ol	alcohols	150.00	green, fruity	0.07 ± 0.01 ^d^	0.96 ± 0.05 ^c^	1.15 ± 0.06 ^b^	1.34 ± 0.03 ^a^	1.27 ± 0.04 ^a^	1.31 ± 0.03 ^a^
14	3-hexanone	ketones	0.06	sweet, fruity, waxy	89.09 ± 8.73 ^f^	294.23 ± 10.73 ^e^	346.08 ± 13.89 ^d^	441.61 ± 10.11 ^c^	488.64 ± 12.88 ^b^	540.24 ± 12.59 ^a^
15	2-butanone	ketones	35.40	pungent, sweet, cheesy	0.56 ± 0.05 ^e^	1.75 ± 0.06 ^d^	2.31 ± 0.10 ^c^	3.58 ± 0.09 ^b^	4.18 ± 0.12 ^a^	4.46 ± 0.14 ^a^
16	2,3-butanedione	ketones	0.18	buttery, caramel-flavored, yogurt-like,	20.65 ± 1.94 ^f^	67.66 ± 2.20 ^e^	99.45 ± 4.62 ^d^	158.3 ± 3.31 ^c^	191.96 ± 6.11 ^b^	210.70 ± 5.95 ^a^
17	2-pentanone	ketones	1.38	sweet, fruity, banana-like	9.99 ± 0.81 ^c^	12.58 ± 0.40 ^a^	10.62 ± 0.48 ^bc^	11.78 ± 0.46 ^ab^	10.01 ± 0.32 ^c^	8.49 ± 0.24 ^d^
18	2-pentyl furan	furan	5.80	fruity, green, bean-flavored	0.15 ± 0.02 ^f^	1.39 ± 0.14 ^e^	2.83 ± 0.19 ^d^	4.19 ± 0.20 ^c^	5.10 ± 0.21 ^b^	5.87 ± 0.17 ^a^

OAV, odor active value. Data are presented as mean ± SEM (n = 6); different small letters indicate significant differences among groups (*p* < 0.05).

## Data Availability

The data are contained within the article.
